# The K-segments of wheat dehydrin WZY2 are essential for its protective functions under temperature stress

**DOI:** 10.3389/fpls.2015.00406

**Published:** 2015-06-11

**Authors:** Wenbo Yang, Linsheng Zhang, Hui Lv, He Li, Yane Zhang, Yang Xu, Jianing Yu

**Affiliations:** ^1^College of Life Sciences/State Key Laboratory of Crop Stress Biology for Arid Areas, Northwest A&F University, YanglingChina; ^2^College of Life Sciences, Shaanxi Normal UniversityXi’an, China

**Keywords:** dehydrin, K-segment, protectant, temperature stress, cell viability, lactate dehydrogenase, protein aggregation

## Abstract

Dehydrins (DHNs), group 2 of late embryogenesis abundant (LEA) proteins, are up-regulated in most plants during cold, drought, heat, or salinity stress. All DHNs contain at least one K-segment, which is believed to play a significant role in DHN function by forming an amphipathic helix. In previous studies, *wzy2*, an YSK_2_-type DHN gene, was isolated from the *Zhengyin 1* cultivar of *Triticum aestivum* under cold and drought stress treatment conditions. Four WZY2 truncated derivatives were constructed to knock out the K-, Y- or S-segment, which potentially affect the function of the protein. *In vivo* assays of *Escherichia coli* viability enhancement, *in vitro* lactate dehydrogenase (LDH) activity protection and *ex vivo* protein aggregation prevention assays revealed that WZY2 acted as a protectant and improved stress tolerance during temperature variation. The results also showed that unlike the truncated derivative without K-segments, the derivative containing two K-segments had remarkable effects that were similar to those of full-length WZY2, indicating that the K-segment is the major functional component of WZY2. Moreover, compared with the other segments, the first K-segment might be the most critical contributor to WZY2 functionality. In general, this work highlights the behavior of DHNs in relieving cold stress *ex vivo* and the contribution of the K-segment to DHN function.

## Introduction

Abiotic stress such as cold, drought, heat, or salinity can cause plant dehydration and even death. As a typical response to such stress, most plants up-regulate the expression of dehydrins (DHNs), which are group 2 of late embryogenesis abundant (LEA) proteins; these proteins are believed to participate in plant environmental stress tolerance (Close, 1997). Most DHNs have one to three repeats of the Y-segment (T/VDEYGNP) near their N-terminus. Following the Y-segment, the S-segment is a tract of five to seven serine residues. The unique characteristic of DHNs is their lysine-rich consensus domain, EKKGIMDKIKEKLPG, which is referred to as the K-segment (Close, 1996) and plays an active role in protecting cellular macromolecules and lipidmembranes (Koag et al., 2009; Drira et al., 2013). It is possible that K-segments do form an amphipathic helix (Allagulova et al., 2003; Hanin et al., 2011). Five types of DHNs have been identified based on the existence of these three segments: Y_m_SK_n_, K_n_, K_n_S, SK_n_, and Y_m_K_n_ (Close, 1996). The Φ-segment, which is also noteworthy, is interspersed throughout DHNs, shows lower conservation and is frequently repeated (Close, 1997). In addition, the DHNs are shown to lack cysteine and tryptophan residues and to be rich in charged and polar amino acids. These properties confer them with highly hydrophilic and boiling-resistant features, similar to other intrinsically disordered proteins (IDPs; Tompa, 2002; Dyson and Wright, 2005).

Some DHNs have been reported to be components of the freezing stress response, including WCOR410 (*Triticum aestivum*; Tsvetanov et al., 2000), COR15 (*Arabidopsis thaliana*; Baker et al., 1994), and CAS15 (*Medicago sativa*; Monroy et al., 1993). Furthermore, it was demonstrated that WCOR410 could improve freezing tolerance of transgenic strawberries (Houde et al., 2004). The α-helical conformation of the K-segment is essential for the binding of DHNs to anionic phospholipid vesicles, as confirmed by lipid vesicle-binding assays of three K-segment deletion derivatives of the maize DHN1 (YSK_2_; Koag et al., 2009). The K-segments of wheat DHN-5 (YSK_2_) protect *Escherichia coli* exposed to various stresses by preventing protein aggregation, and DHN-5 also acts as an antibacterial and antifungal factor during biotic stress (Drira et al., 2015). Moreover, DHN-5 was found to protect lactate dehydrogenase (LDH), β-glucosidase, and glucose oxidase from cold and heat damage *in vitro* (Brini et al., 2010; Drira et al., 2013). Interestingly, truncated forms of DHN-5 with one or two K-segments also showed the same function, whereas YS-truncated derivatives had no effect in these experiments (Drira et al., 2013). Two variants of YSK_2_-type VvcDHN1a have been reported: spliced DHN1a_s (YSK_2_) and unspliced DHN1a_u (YS). Only DHN1a_s was reported to be involved in resistance to cold and drought as well as the growth of *Botrytis cinerea* (Rosales et al., 2014). There are two forms of DHN in *Jatropha curcas* seeds, JcDHN_1 (Y_3_SK_2_) and JcDHN_2 (Y_2_SK_2_); the transcript level change of JcDHN_2 was 8-fold that of JcDHN_1 at its maximum value, a time when the water content of the seed changed dramatically from 42% for mature seeds to 12% for desiccated seeds (Omar et al., 2013). In addition, LEA proteins might function as molecular chaperones (Wise and Tunnacliffe, 2004) to help non-natural proteins resist aggregation *in vitro* (Goyal et al., 2005).

In previous studies, we cloned the full-length cDNA of the DHN *wzy2* (accession no. EU395844, YSK_2_) from the wheat cultivar *Zhengyin 1*. *wzy2* expression varies depending on genotype, stress type, and stress duration (Huang et al., 2009; Zhu et al., 2012). Furthermore, quantitative real-time PCR analysis of *wzy2* showed that this gene could be induced by low temperature, anoxia, indoleacetic acid, methyl jasmonate, abscisic acid, and gibberellin treatments (Zhu et al., 2014).

To further understand the function of the K-segment of DHNs, we generated four truncated WZY2 constructs; each construct retained different conservative segments of the protein. Our data provide evidence that the K-segment plays a significant role in WZY2 function. This segment is critical for maintaining bacterial growth, enhancing LDH activity, and preventing protein aggregation during temperature stress.

## Materials and Methods

### Construction, Expression, and Purification of WZY2 and its Truncated Derivatives

Full-length and truncated *wzy2* cDNAs were amplified with specific primers (Supplementary Table S1). All the forward primers contained an *Eco*R I site (underlined), and the reverse primers contained a *Hind* III site (underlined). PCR reactions to achieve the desired Y-, S-, and K-segment deletions were performed following the protocol for overlap extension PCR (Heckman and Pease, 2007) using TransStart FastPfu DNA Polymerase (Transgen, P.R. China). The open reading frame (ORF) of *wzy2* was amplified from the plasmid *wzy2-*pMD19-T with the wt_F/wt_R primers (Junyi et al., 2012). The PCR products were digested with *Eco*R I and *Hind* III and ligated into the expression vector pET28a (Novagen, USA), which had been digested with the same enzymes. The recombinant plasmid was transformed into *E. coli* strain BL21 (DE3) according to Novagen’s protocol. The plasmid *wzy2-*pET28a, containing the full-length *wzy2* gene cDNA, was used as the template for the generation of the truncated derivatives.

**FIGURE 1:**
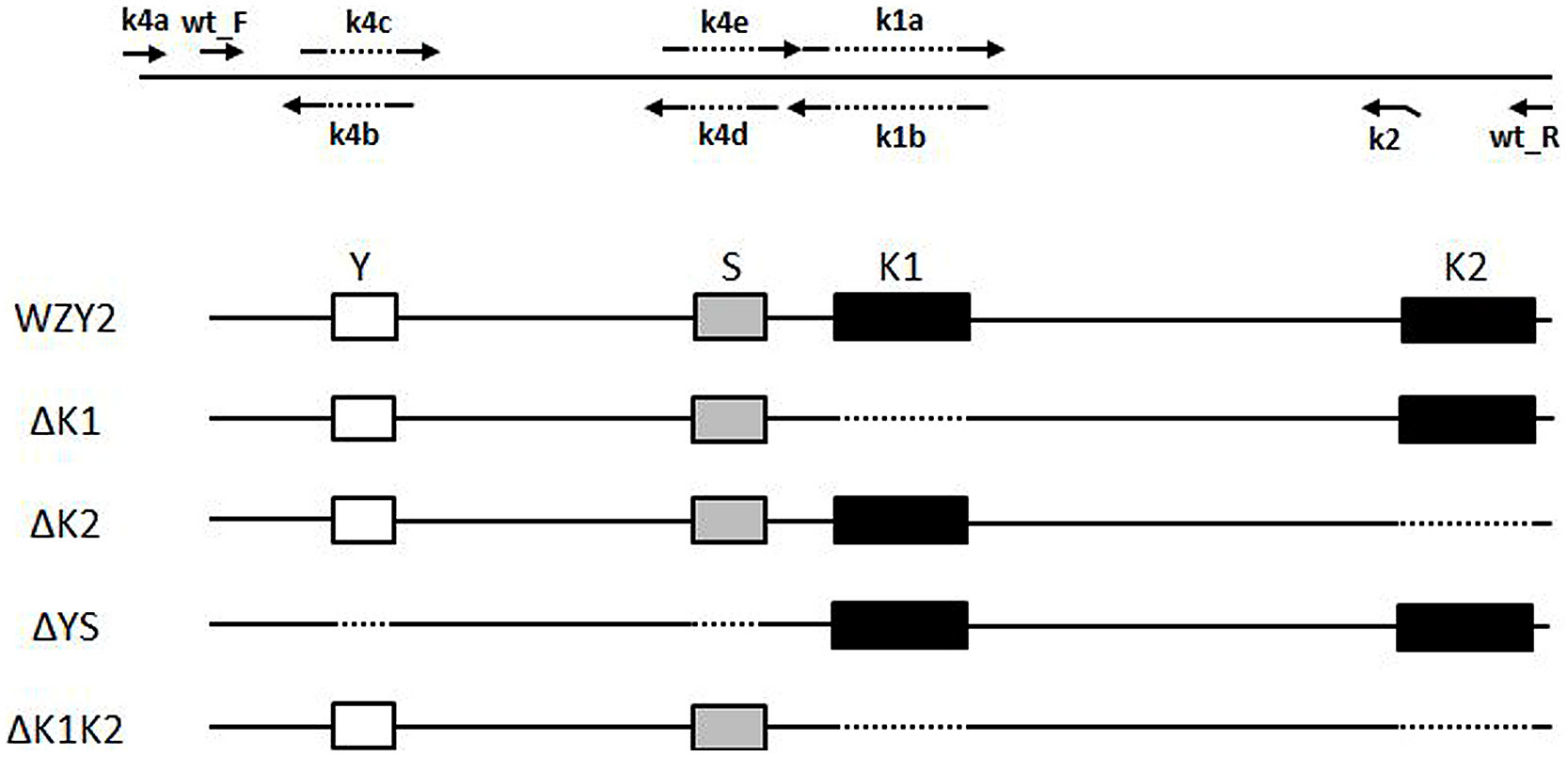
**Schematic representation of the diverse segments of WZY2.** The positions of the conserved segments Y (white box), S (gray box), and K (black box), and the PCR primers are noted. The dotted line indicates the deleted sequences.

To obtain recombinant proteins without the specified segments (i.e., ΔK1, ΔK2, ΔYS, and ΔK1K2), eight primers were designed and employed in the PCR protocol. Some primers included the nucleotide sequence that spanned the region targeted for deletion (**Figure 1**). The first K-segment deletion variant (ΔK1) was generated with primers wt_F/wt_R; the amplified product mixture of wt_F/k1b and k1a/wt_R (amplified from *wzy2-*pET28a) at a 1:1 ratio as the template for this reaction. To remove the second K-segment (K2) to obtain ΔK2, primers wt_F/k2 were used. To generate the variant ΔK1K2 lacking both K-segments, PCR was performed with primers wt_F/k2. The ΔK1 PCR amplified fragments were used as the template. The strategy to generate variant ΔYS, with deleted Y- and S-segments, was based on *wzy2-*pET28a. Three pairs of primers (i.e., k4a/k4b, k4c/k4d, and k4e/wt_R) were utilized to generate three small products from this plasmid, and the mutant protein ΔYS was created by splicing these pieces together. All PCR products were inserted into the pET28a vector following digestion with *Eco*R I and *Hind* III, and the vector was transformed into *E. coli* BL21 (DE3). All recombinant constructs were confirmed by DNA sequencing.

*Escherichia coli* BL21 (DE3) strains carrying recombinant plasmids WZY2, ΔK1, ΔK2, ΔYS, or ΔK1K2 or the control vector pET28a were grown in Luria-Bertani (LB) medium with 50 μg/mL kanamycin at 37°C until an OD600 = 1.0 was reached. Protein expression was induced by 1 mM isopropyl β-D-thiogalactoside (IPTG) for 3 h. After the induction period, the bacteria were harvested by centrifugation at 6,000 × *g* for 10 min at 4°C, and the pelleted cells were suspended with phosphate-buffered saline (PBS; pH 7.0). The *E. coli* cells suspended in PBS were placed in liquid nitrogen for 5 min and then successively incubated in a 90°C water bath for 30 min with manual agitation every 10 min. The upper clear supernatant was transferred to a fresh tube after centrifugation at 12,000 × *g* for 15 min at 4°C, and a second centrifugation step was required. Soluble proteins were purified with a gel filtration system with Sephacryl S-100 High Resolution medium (GE Healthcare, USA) and verified by gel electrophoresis.

### *E. coli* Stress Tolerance Assay

To evaluate temperature stress tolerance, 1 mL of culture medium from each IPTG-induced sample was taken, and the samples were adjusted to equal cell concentrations. The samples were incubated at different temperatures (0, 37, and 50°C) for 20 min before being diluted at an appropriate ratio for spotting onto LB basal plates. Cell survival was measured as the ratio of colony-forming units (CFUs) between the treatment (0 or 50°C) and control (37°C). The experiment was repeated three independent times in triplicate for each sample.

### LDH Protection Analysis

Lactate dehydrogenase activity was used as a marker to evaluate the protective function of DHN during cold and heat stresses. The assay was based on that described by Drira et al. (2013), with minor modifications. LDH (EC1.1.1.27, from rabbit muscle, Sigma-Aldrich, USA) was dissolved in 10 mM sodium phosphate (pH 7.4) at a final concentration of 10 μg/mL. Purified WZY2, its truncated variants, HIS (from the control vector), and bovine serum albumin (BSA, Sigma-Aldrich, USA) were all prepared at 20 μg/mL with 10 mM sodium phosphate (pH 7.4). The LDH assay reaction buffer contained 2 mM NADH, 10 mM pyruvic acid, and 10 mM sodium phosphate (pH7.4). Equal volumes of LDH enzyme solution and protein solution were mixed for temperature stress treatments. For the cold stress test, we incubated LDH and the protein mixture on ice for 0, 2, 4, 6, 8, 10, 12, or 24 h and transferred 70 μL of the mixture into 630 μL of reaction buffer at the end of the incubation. After thorough mixing, the samples were incubated at 25°C for 30 s, and the absorbance at 340 nm was measured at 30-s intervals for 6 min using a microplate reader (SpectraMax M2, Molecular Devices, USA). Triplicate reactions were set up for each treatment. The LDH reaction data were analyzed with ancillary software (SoftMax Pro, Molecular Devices, USA). A high temperature stress test was also performed by incubating the LDH and protein sample mixtures in a 45°C water bath and adding 70–630 μL of the reaction buffer at 0, 10, 20, and 30 min. The LDH activity was then measured as in the cold stress test.

### Plant Materials and Growth Conditions

The winter wheat (*T. aestivum* L.) cultivar *Zhengyin 1*, from which *wzy2* was first isolated, was used in this study. Seeds were obtained from the College of Life Science of Northwest A&F University in Yangling, China. The plants were grown in a commercial soil mix under 200 μE m^-2^s^-1^ light at 18/25°C (night/day) temperature with a 16-h photoperiod.

### Wheat Leaf Blade Epidermal Cell Transient Expression Assay

The vector pTF486, containing cauliflower mosaic virus (CaMV) 35S promoter-driven enhanced GFP (eGFP), was used in this study (Yu et al., 2008). To create a C-terminal GFP-tagged fusion protein, the ORF of *wzy2* was amplified using primers GFP_F and GFP_R1 (Supplementary Table S1) and then inserted into the pTF486 vector at the *Nco* I site (underlined) to generate the recombinant plasmid P35S::WZY2::GFP. The same set of primers was used to generate the constructs P35S::ΔK1::GFP and P35S::ΔYS::GFP. P35S::ΔK2::GFP and P35S::ΔK1K2::GFP, which were created using primers GFP_F and GFP_R2 (Supplementary Table S1). All the generated recombinants were confirmed by DNA sequencing and prepared for transient expression in wheat leaf blade epidermal cells. The desired plasmids were introduced into the host cells using a gene gun.

Young leaves of *Zhengyin 1* at the third leaf stage were selected and disinfected with 70% ethyl alcohol for 10 s, followed by rinsing three times with sterile water. These disinfected leaves were then snipped to 3-cm-long pieces and grown on Murashige and Skoog (MS) solid medium at 25°C for 4 h in the dark. Five constructs and a control vector were used for transfection via the PDS-1000/He gene gun system (Bio-Rad, USA), per the manufacturer’s protocol. After 24 h of incubation, the wheat leaf tissues were kept at 4°C for 72 h and then rewarmed at 25°C for 24 h. GFP fluorescence in the transfected wheat leaf blade epidermal cells was examined periodically (at 4°C at 0, 12, 24, 48, and 72 h, and at 25°C at 12 h and 24 h) during the incubation using a Leica DM5000 fluorescence microscope system (Germany).

### Statistical Analysis

All data were analyzed with a *t*-test or two-way ANOVA using GraphPad Prism version 5.0 (USA). Significant differences were considered at a *P*-value less than 0.05.

## Results

### Sequence Analysis of WZY2 and its Truncated Derivatives

To determine whether the K-segment is necessary for WZY2, we constructed a series of truncated recombinant WZY2 proteins (**Figure 1** and **Supplementary Figure S1**). Full-length WZY2 contains a Y-segment (Y), an S-segment (S), and two K-segments (K1, K2). The derivative proteins ΔK1 and ΔK2 had either the first K-segment (K1) or the second K-segment (K2) deleted. ΔYS lacked the Y plus S elements but retained both K-segments. For ΔK1K2, the K1- and K2-segments were removed, but the Y plus S segments were retained, in contrast to ΔYS. DNA sequencing was used to verify that the mutants were only deleted for the selected regions.

Dehydrins are considered IDPs (Hughes and Graether, 2011), which do not have a unique, well-defined protein structure (Hughes et al., 2013). Using the disordered region prediction tool PONDR-Fit^1^, the intrinsically disordered characteristics of these full-length, and truncated proteins were calculated (Dunker et al., 2002); the scores were all higher than 0.5, indicating that all five proteins are disordered. There were three low-score fragments in the amino acid sequence of WZY2: 20–50, 70–90, and 125–150, corresponding to the regions between the Y- and S-segments, K1-segment, and K2-segment, respectively (**Supplementary Figure S2**). A consistent conclusion could be inferred from the curves of the WZY2 truncated derivatives. Moreover, the curve obtained for ΔYS was similar to that of WZY2, except for a slight decrease in the first low-score region (**Supplementary Figure S2**).

**FIGURE 2:**
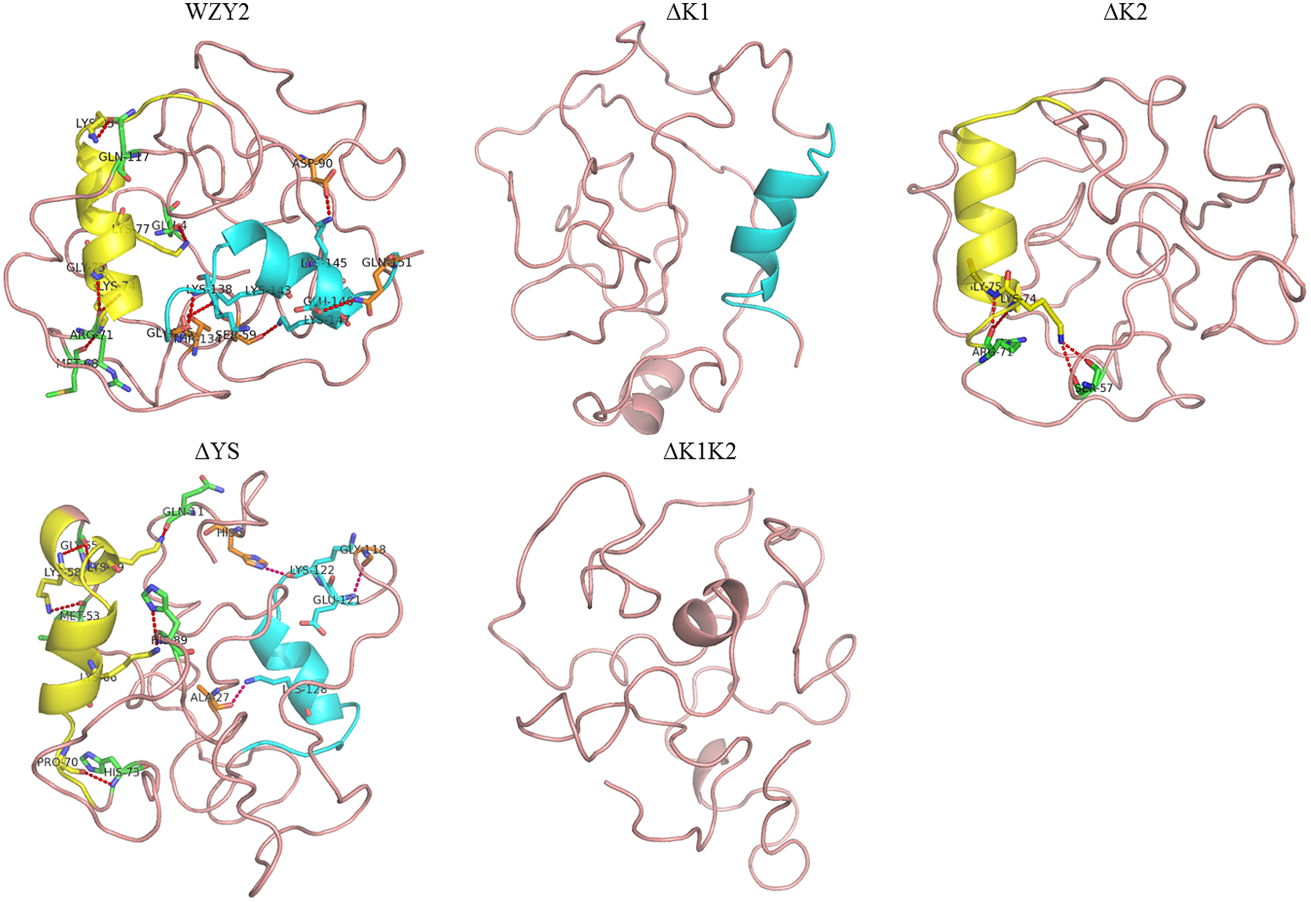
**Three-dimensional structure prediction of WZY2 and its truncated derivative polypeptides.** The K1-segment is shown in yellow, and the K2-segment is in blue. The amino acid residues involved in the helices-protein interaction are shown in yellow (in the K1-segment), in green (with the K1-segment), in blue (in the K2-segment), and in orange (with the K2-segment).

The 3D structures of WZY2 and its truncated derivatives were predicted using Phyre2^2^. As expected, WZY2 was predicted to contain the random coil and two α-helices related to the K-segments. All nine amino acid residues involved in the helices-protein interaction were polar amino acids, and seven of them were lysines (**Figure 2**). ΔYS also showed two α-helices and seven polar amino acids, including five lysines that involved in interactions between helices and protein (**Figure 2**). ΔK1 and ΔK2 contained one α-helix each, with only two residues (ΔK2) in the interaction (**Figure 2**). ΔK1K2 was predicted to adopt a loosely folded structure with a random order (**Figure 2**).

### WZY2 and its Truncated Derivatives Enhanced the Viability of *E. coli* Cells Under Freezing and Thermal Stress Conditions

To investigate whether WZY2 and its truncated derivatives enhance stress tolerance *in vivo*, the cell viability of *E. coli* transformed with WZY2, ΔK1, ΔK2, ΔYS, ΔK1K2, or the control vector was measured under freezing and thermal conditions. These cultures were treated at 0, 37, or 50°C, as described earlier in Section “Materials and Methods,” and survival ratios were calculated. A temperature of 37°C was chosen as the standard condition, and the control vector was used as a negative control.

**FIGURE 3:**
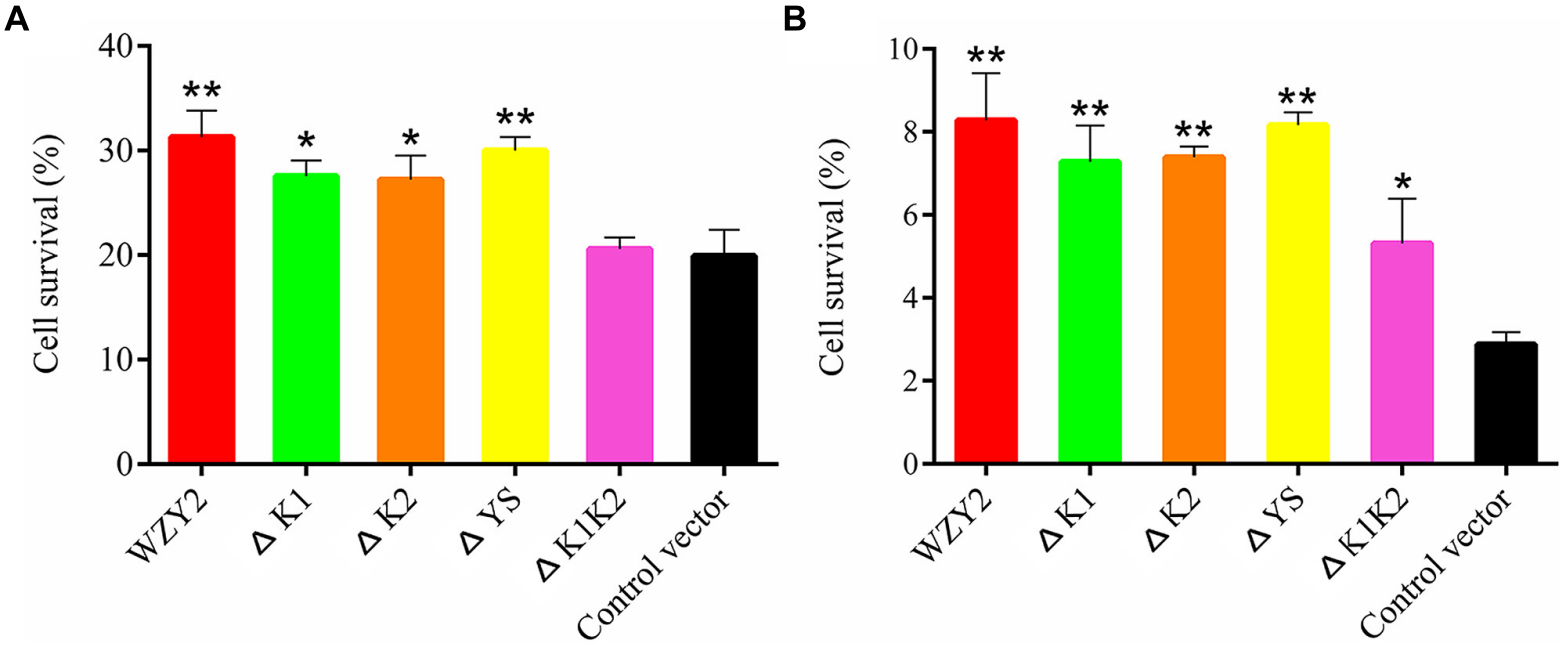
**The cell viability of *Escherichia coli* transformed with WZY2, ΔK1, ΔK2, ΔYS, and ΔK1K2 or the control vector when subjected to temperature stress.** *E. coli* cells were exposed to 0°C **(A)** or 50°C **(B)** for 20 min, and CFUs were calculated. The cell survival ratio was derived from a comparison of CFUs at 37°C. Three independent assays were performed, and SE are included. Values are the mean ± SD (*n* = 3). Significant differences in the survival ratio are indicated as *P < 0.05 or **P < 0.01, which were evaluated with a *t*-test.

The bacterial cells overexpressing WZY2 and ΔYS showed the best viability under cold stress, followed by ΔK1 and ΔK2. The growth of *E. coli* with ΔK1K2 or the control vector was ~10% lower than that of the others (**Figure 3A** and Supplementary Table S2). The survival rates of the cells overexpressing WZY2, ΔYS, ΔK1, ΔK2, and ΔK1K2 were up to 2.8-, 2.8-, 2.5-, 2.5-, and 1.8-fold, respectively, compared with the controls after heat treatment (**Figure 3B**). In both stress treatments, ΔYS, similar to WZY2, showed a better effect on improving viability than did ΔK1K2. These data confirmed that the K-segments of WZY2 are crucial for the temperature tolerance of *E. coli*.

### WZY2 and its Truncated Derivatives Protected LDH Enzyme Activity Under Freezing and Thermal Stress Conditions

Because LDH loses its activity during freezing and thermal conditions, we selected this enzyme to evaluate the protective effect of WZY2 and its truncated derivatives. The time-dependent loss of LDH activity was measured as described previously in a method that employed BSA and PBS buffer as the positive and negative controls, respectively. WZY2 and its truncated derivatives were purified by a gel filtration system and verified by sodium dodecyl sulfate polyacrylamide gel electrophoresis (SDS-PAGE; **Supplementary Figure S3**). LDH activity before treatment was regarded as 100%.

**FIGURE 4:**
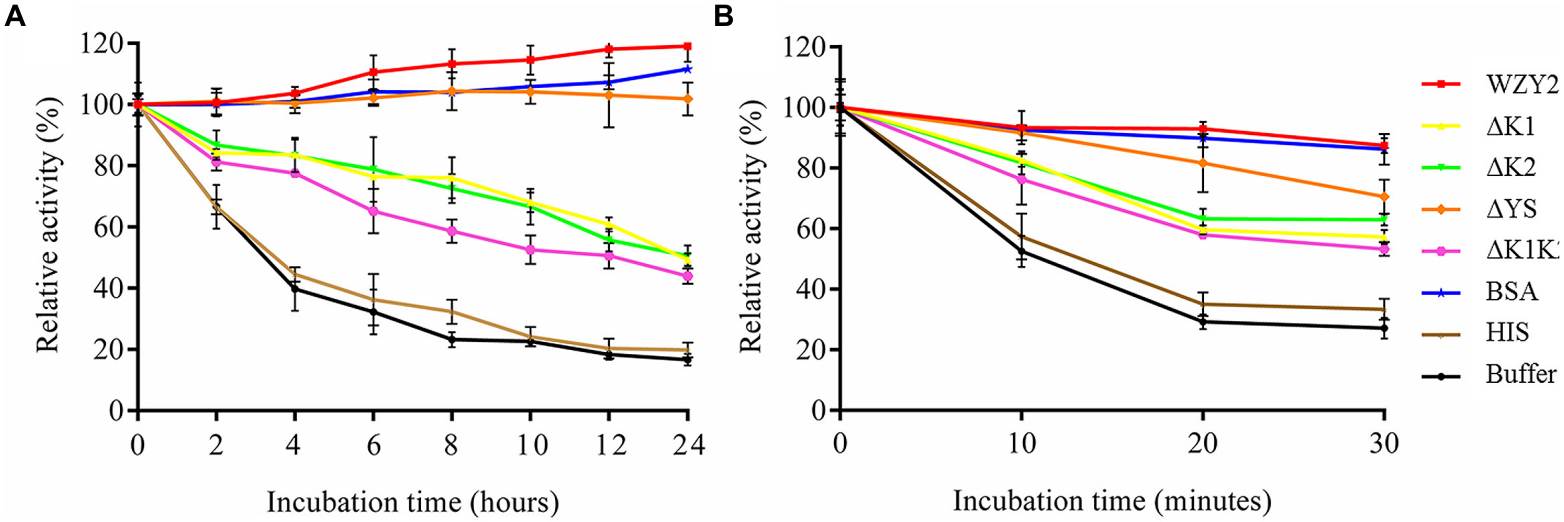
**WZY2 and its truncated derivatives protected LDH activity from freezing and thermal stress. LDH solutions in the presence of BSA, HIS, and purified recombinant proteins WZY2, ΔK1, ΔK2, ΔYS, and ΔK1K2 were incubated on ice **(A)** or at 45°C **(B)** for the specified times. LDH activity was measured at each particular time point. The results are expressed as the LDH activity (%) relative to the activity prior to treatment. Three independent assays were performed, and SE are included. Values are the mean ± SD (*n* = 3)**.

After incubation in an ice-bath for 24 h, HIS did not show any protective effect on LDH, with rates of activity loss that were similar to the buffer condition (**Figure 4A**). In contrast, LDH activity increased nearly 20% in the presence of WZY2, which was higher than that with BSA (12%; **Figure 4A**). ΔYS exhibited a similar protective effect to that of BSA, and the activity rates reached 102% up to the end of the freezing stress (**Figure 4A**). When LDH was incubated on ice in the presence of ΔK1, ΔK2, or ΔK1K2, the activities showed the same trend, decreasing to ~45–50%. After 4 h of incubation with ΔK1 or ΔK2, LDH still retained more than 80% of its activity, and this retention was much longer than that observed for ΔK1K2 (2 h; **Figure 4A**).

The effects of WZY2 and its truncated derivatives with regard to protecting LDH activity during heat stress were also evaluated. In the first 10 min, LDH activity with HIS and buffer decreased nearly 50%, with only 30% remaining after 30 min (**Figure 4B**). In contrast, LDH inactivation was dramatically reduced in the presence of WZY2, with 87% of the activity being retained after 30 min (**Figure 4B**); WZY2 also protected LDH activity more effectively than BSA. The protective effects of the truncated derivatives on LDH activity resulted in similar rates of enzyme activity; among the truncated derivatives, ΔYS and ΔK1K2 showed the highest and lowest protection efficiency, respectively (**Figure 4B**). The protective effects of WZY2 and ΔYS were similar to each other, and these proteins offered greater protection than the other truncated forms; ΔK1, ΔK2, and ΔK1K2 were also similar to each other in terms of their protective effects and offered a moderate level of protection.

### WZY2 and its Truncated Derivatives Prevented Protein Aggregation Under Cold and Rewarming Conditions

**FIGURE 5:**
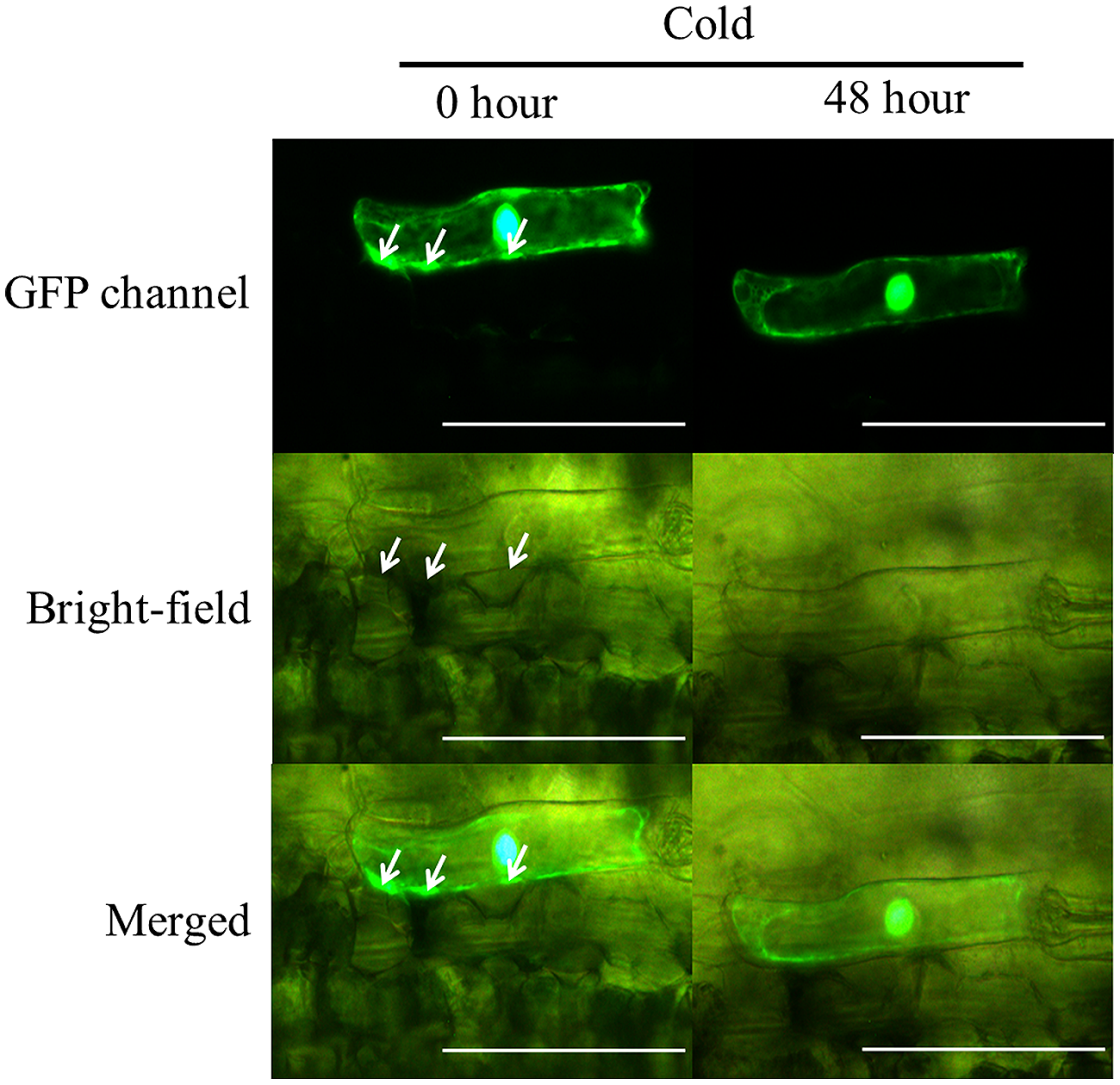
**Subcellular localization of WZY2::GFP fusion proteins.** The localizations of WZY2::GFP at 0 h and 48 h under cold treatment are shown in the images from the GFP channel, bright-field and merged images. The bar indicates 100 μm. Green fluorescence was dispersed in the nucleus, interspersed in the cytoplasm, and accumulated in spots (white arrow) near the plasma membrane.

The WZY2 (or ΔK2, ΔYS, ΔK1K2)::GFP fusion protein was overexpressed in *Zhengyin 1* leaf epidermal cells (ΔK1::GFP could not be observed), and GFP fluorescence was examined by fluorescence microscopy. All fusion proteins and GFP were observed in the nucleus and cytoplasm prior to low temperature stress. In dehydrated cells, some green fluorescence spots (white arrow in **Figure 5**) were found near the plasma membrane where the fusion proteins aggregated. The pattern of aggregation varied during cold and rewarming treatments.

Control GFP diffused throughout the entire cell, and this pattern did not change with an increase or decrease in temperature. The accumulated WZY2::GFP and ΔYS::GFP proteins disappeared on the third day of cold stress (**Supplementary Figure S4**). ΔK2::GFP was found to diffuse from the spots after 48 h of cold treatment, and the aggregation dissipated within 24 h of rewarming (**Supplementary Figure S4**). The distribution of ΔK1K2::GFP showed no change compared to controls during the entire treatment, similar to GFP (**Supplementary Figure S4**). The merged image of the GFP channel and bright field for WZY2::GFP showed that the fusion proteins were located adjacent to the space between the epidermal cells and mesophyll cells before cold stress. No fusion proteins were found in this area after 48 h of cold treatment, even though plasmolysis was occurring (**Figure 5**). To maintain the consistency of the experiment, each fusion protein was observed in one cell, and images were captured under the same conditions.

## Discussion

Dehydrins are a group of intrinsically disordered proteins that respond to abiotic and biotic stresses (Graether and Boddington, 2014; Rosales et al., 2014). The importance of DHNs has been demonstrated by genetic and protein evidence *in vitro*; however, an *ex vivo*, or *in vivo* protective mechanism has not been elucidated (Graether and Boddington, 2014).

The wheat DHN WZY2 is induced by drought, low temperature, and other stresses (Zhu et al., 2014). The data presented in the present paper indicate that WZY2 exerts its function as a protectant. It increases *E. coli* viability, protects LDH activity and inhibits protein aggregation during temperature variation. The K-segment is a major functional component of this DHN. The proteins with two K-segments (WZY2 and ΔYS) had the most significant impact on improving cold and heat stress tolerance in all the experimental systems employed in this study. ΔYS performed in a manner similar to WZY2 with regard to maintaining bacterial growth, enhancing LDH activity and preventing protein aggregation. The recombinant proteins that contained one K-segment (i.e., ΔK1 and ΔK2) had complex effects, and ΔK1 and ΔK2 had similar effects that were smaller than those of WZY2 and ΔYS both *in vitro* and in *E. coli* cells. ΔK1::GFP could not be observed in wheat leaf epidermal cells under fluorescence microscopy. Although the recombinant protein with no K-segment (ΔK1K2) exhibited higher LDH activity protection efficiency than the controls, it resulted in almost identical consequences as the controls in other assays.

In our study, K1, K2, Y plus S, or K1 plus K2 were removed from full-length WZY2 to generate the truncated derivatives ΔK1, ΔK2, ΔYS, and ΔK1K2, respectively. DNA sequence analysis verified that except for the removed component(s), the remainder of the proteins remained intact in the constructs. Since no additional amino acids was removed, ΔK1K2 differed from the recombinant protein YS (from TaDHN5; Drira et al., 2013) in protecting LDH activity under freezing and thermal stresses.

Group 1 and group 3 LEA proteins have been shown to function as molecular chaperones that protect citrate synthase and LDH from aggregation due to water stress (Goyal et al., 2005). LEA proteins are not classical chaperones but are more likely unstructured proteins, such as α-synuclein (Kumar et al., 2014; Manda et al., 2014) and *Eh*PDI (Mares et al., 2014). Chaperones will translocate to specific locations to carry out their functions (Vaseva et al., 2012), and DHNs behave in the same way. To provide the most realistic intracellular microenvironment for WZY2, wheat *Zhengyin 1* leaf tissues were used, and single cells were selected to measure each recombinant protein. This work tested the functions of protective agents in *ex vivo* experimental systems for the first time and further evaluated the results of other experiments *in vitro* (LDH activity) and *in vivo* (bacterial viability).

Previous studies on dynamic changes in DHN protein localization in cells were performed using immunohistochemical methods. Immuno-gold labeling of LTI29 (*A. thaliana*) indicated that this protein changed location from the cytoplasm to the plasma membrane in cold acclimated (Puhakainen et al., 2004). Comparing immunomicroscopy images of wheat *Irkutskaya ozimaya* seedlings grown at 4 and 22°C, it was clear that the density of DHNs increased significantly in the rough endoplasmic reticulum, mitochondria, and intercellular space at low temperatures (Romanenko et al., 2010). One paper reported dynamic DHN movement in protoplasts. *Actinidia chinensis* DHN1 is normally located in the nucleus and then moves to the cytoplasm near the plasma membrane in response to osmotic stress (Qiu et al., 2001). Based on available evidence, DHNs move toward the plasma membrane and intracellular membrane systems during cold acclimation, a result that is consistent with DHNs’ binding gain of membrane stability *in vitro* (Koag et al., 2003, 2009; Rahman et al., 2010, 2011a,b; Rahman, 2012). However, our results conflict with this notion. Indeed, WZY2, ΔYS, and ΔK2 left areas near the plasma membrane under cold stress. This result indicated that unlike the DHNs mentioned above, the primary function of WZY2 is to protect biomacromolecules and prevent protein aggregation.

Analysis of the circular dichroism (CD) spectra of DHN1 (YSK_2_) and its truncated proteins with anionic lipid vesicles showed that wild-type DHN1 and ΔK2 (the deletion mutant lacking the second K-segment) adopt more α-helical structures than ΔK1 (the deletion mutant lacking the first K-segment; Koag et al., 2009). The comparison of the CD spectra of mutant proteins with K-segment deletions in the presence of SDS showed that ΔK2, but not ΔK1, was nearly equivalent to normal DHN1 (Koag et al., 2009). It should be noted that we could not observe the ΔK1 recombinant in wheat leaf epidermal cells. Our hypothesis is that ΔK1::GFP may be an unstable protein in wheat leaf epidermal cells or that the first K-segment may be the most critical segment in this wheat expression system, with its deletion leading to reduced or no WZY2 expression. This speculation requires further investigation.

The procedure for monitoring dynamic changes in the localization of GFP-tagged DHNs in our system was rapid and easy to perform and showed the natural movements of the DHNs. However, not all stresses were suitable, such as the high temperature stress, because the leaf tissue could not survive for 48 h at high temperatures. It would be worthwhile to apply this method to the study of osmotic, salinity, and heavy-metal ion stresses. The DHNs showed different behaviors in different stress situations with regard to factors such as the pathway and strength of dynamic movement. If the movement of DHNs is predictable, these proteins will be potential candidates for the experimental systems employed in this study.

The data represented in this paper strongly suggest that WZY2 is essential for the maintenance of the cell survival rate, the protection of LDH enzyme activity, and the prevention of protein aggregation under temperature stress. The results illustrate that K-segments play a significant role in WZY2 function, especially the first K-segment. Further studies are needed to determine which proteins regulated by WZY2, how WZY2 protects proteins from aggregating *ex vivo* and *in vivo*, and the contribution of the K1-segment to the function of WZY2.

## Author Contributions

WY proposed the ideas and designed the experiments. WY, HL, HL, and YX performed the experiments. WY and YZ analyzed the data. WY drafted the manuscript and revised it. JY and LZ ensured that all work was appropriately investigated. All authors approved the final manuscript.

## Conflict of Interest Statement

The authors declare that the research was conducted in the absence of any commercial or financial relationships that could be construed as a potential conflict of interest.

## Footnotes

1 http://www.disprot.org/pondr-fit.php

2 www.sbg.bio.ic.ac.uk/phyre2

## Supplementary Material

Figure S1 **Comparison of the amino acid sequences of full-length WZY2 and the deletion mutants.** The primary amino acid sequences and Y-, S-, and K-segments are shown. Hyphens indicate the deleted positions.

Figure S2 **Disoons predicted with PONDR-Fit software for WZY2 and its truncated derivative polypeptides.** Green lines represent the confidence scores of the disorder prediction. Predictions were evaluated with a standard, such that scores of 0.0–0.5 indicated order and 0.5–1.0 indicated disorder.

Figure S3 **SDS-PAGE of purified WZY2 and its truncated derivatives expressed in BL21.** Proteins were loaded in the order of marker (lane 1), HIS (control vector, lane 2), WZY2 (lane 3), ΔK1 (lane 4), ΔK2 (lane 5), ΔYS (lane 6), and ΔK1K2 (lane 7). Protein sizes in kDa are shown to the left of the panel.

Figure S4 **Subcellular localization of WZY2 (or ΔK2, ΔYS, ΔK1K2)::GFP fusion proteins.** WZY2 (or ΔK2, ΔYS, ΔK1K2)::GFP fusion protein subcellular localization varied in wheat leaf blade epidermal cells when subjected for specified times to cold and rewarming treatments. The bar indicates 100 μm. Green fluorescence was dispersed in the nucleus, interspersed in the cytoplasm, and accumulated in spots (white arrow) near the plasma membrane.
