# Supplementary material for: The K-segments of wheat dehydrin WZY2 are essential for its protective functions under temperature stress
Source: Front Plant Sci. 2015 Jun 11;6:406. doi: 10.3389/fpls.2015.00406 (PMC4467595; doi:10.3389/fpls.2015.00406)
Supplement: Supplementary file 1 [file Table_1.PDF]

Supplementary Table 1. Primers used in generating recombinant *wzy2* and its truncated derivatives.

Primer name	Primer sequence
wt_F	5'-CCGGAATTCATGGAGTACCAGGGACATCAGCAGCACGGT-3'
wt_R	5'-CCCAAGCTTTCAGTGCTGTCCGGGCAGCTTCTCCTT-3‘
k1a	5'-GATGATGGCATGGGCGGGAGGGGCCACGGCGAC-3'
k1b	5'-CTGCTGCTGGTCGCCGTGGCCCCTCCCGCCCATGCCATCATC-3'
k2	5'-CCCAAGCTTTCAGTGCTGGCCGGTGCCGTCGGTGC-3'
k4a	5'-ATGCGTCCGGCGTAGAGGATCGAGATCTCGATCCCG-3'
k4b	5'-ATGTCCGGCAACCGGGTTAGTCGCCTGACCGTG-3'
k4c	5'-GGTCAGGCGACTAACCCGGTTGCCGGACATGGC-3'
k4d	5'-CATGCCATCATCCTCGCCGGAGCGCTGCAGGAT-3'
k4e	5'-CTGCAGCGCTCCGGCGAGGATGATGGCATGGGC-3‘
GFP_F	5'- CATGCCATGGAGTACCAGGGACATCAGCAGCACGGTCAGGCG-3'
GFP_R1	5'- CATGCCATGGAACCACCACCACCACCGTGCTGTCCGGGCAG-3'
GFP_R2	5'- CATGCCATGGAACCACCACCACCACCGTGCTGGCCGGTGCC-3'

Restriction sites are underlined.
