# Supplementary material for: The K-segments of wheat dehydrin WZY2 are essential for its protective functions under temperature stress
Source: Front Plant Sci. 2015 Jun 11;6:406. doi: 10.3389/fpls.2015.00406 (PMC4467595; doi:10.3389/fpls.2015.00406)
Supplement: Supplementary file 2 [file Table_2.PDF]

Supplementary Table 2. The growth performance of recombinants WZY2, ΔK1, ΔK2, ΔYS, and ΔK1K2 and the control vector in cell viability assays of *E. coli* transformants at different temperatures.

	Colony-forming units								
Vector	0ºC			50ºC			37ºC		
WZY2	128	137	150	31.6	41.6	36.6	474	396	456
ΔK1	137	99	112	25.95	31.5	32.75	474	372	406
ΔK2	125	117	138	33.2	34.4	35.6	536	468	392
ΔYS	153	161	148	43.7	40.7	41.3	480	512	548
ΔK1K2	112	112	102	23.3	26.6	34.3	560	496	530
Control vector	122	118	96	16.2	17.8	14.6	576	548	552
