# Supplementary figures and images for: The K-segments of wheat dehydrin WZY2 are essential for its protective functions under temperature stress
Source: Front Plant Sci. 2015 Jun 11;6:406. doi: 10.3389/fpls.2015.00406 (PMC4467595; doi:10.3389/fpls.2015.00406)

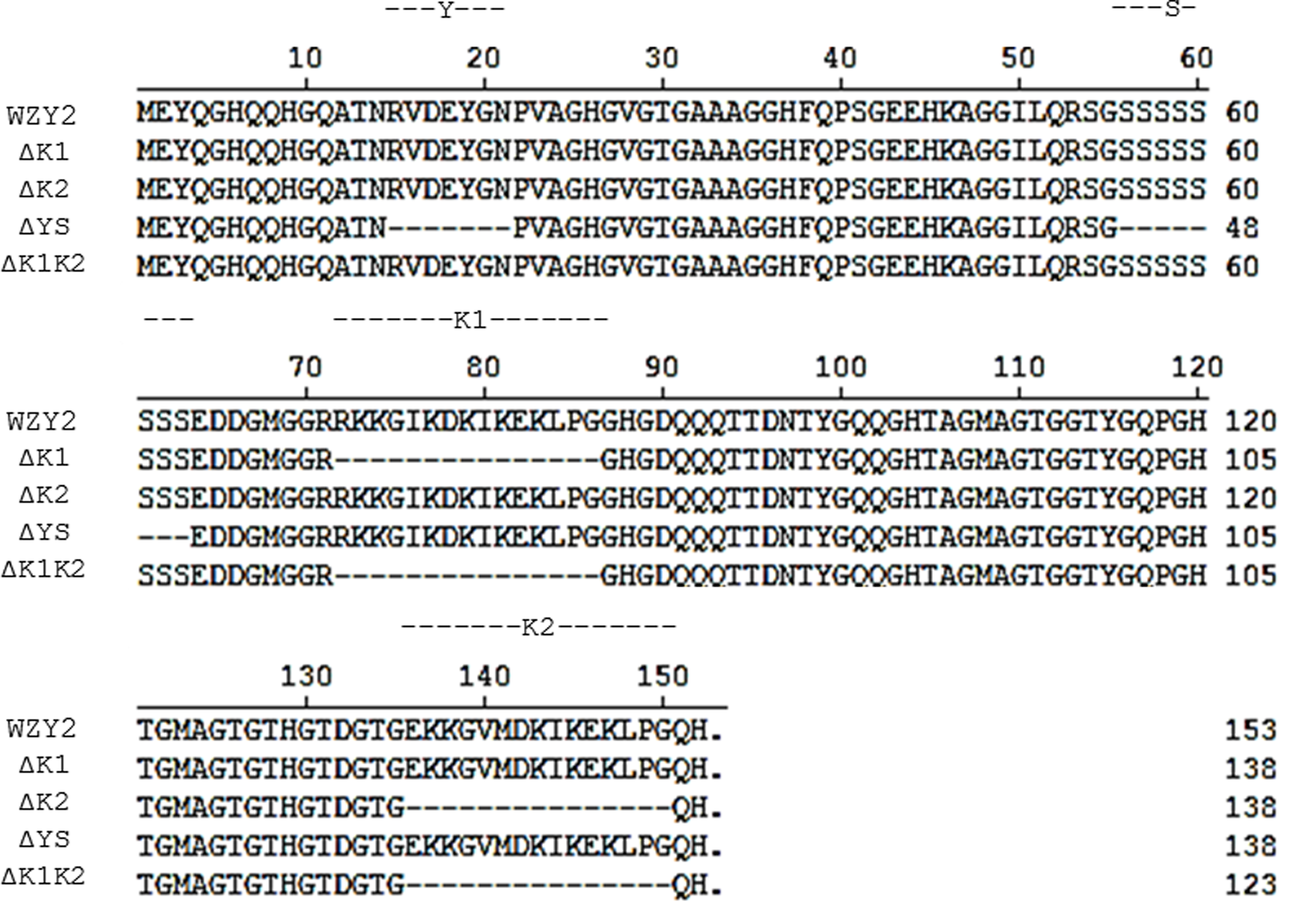

Supplement: Figure S1 — Comparison of the amino acid sequences of full-length WZY2 and the deletion mutants. The primary amino acid sequences and Y-, S-, and K-segments are shown. Hyphens indicate the deleted positions. [file Image_1.TIF]

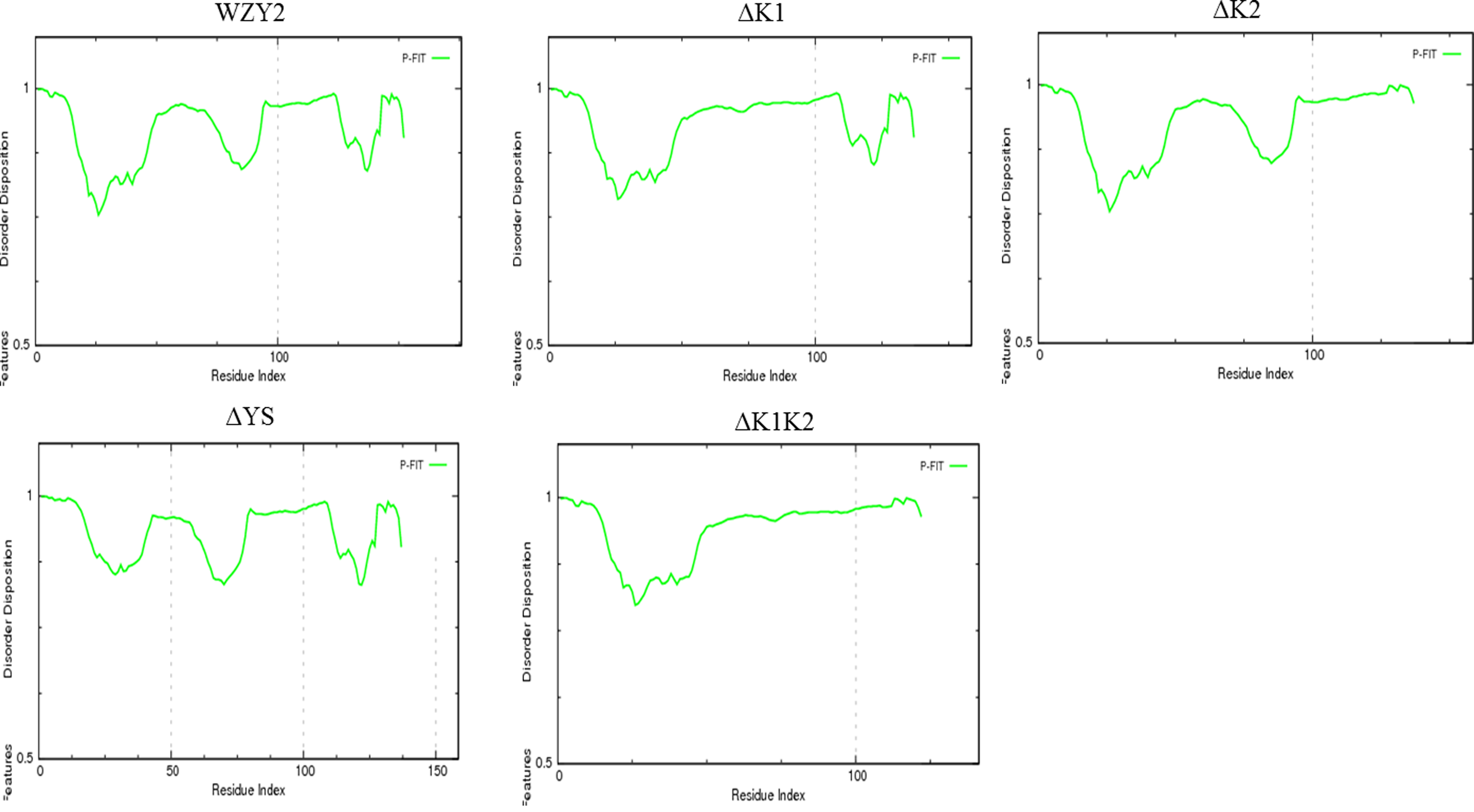

Supplement: Figure S2 — Disoons predicted with PONDR-Fit software for WZY2 and its truncated derivative polypeptides. Green lines represent the confidence scores of the disorder prediction. Predictions were evaluated with a standard, such that scores of 0.0–0.5 indicated order and 0.5–1.0 indicated disorder. [file Image_2.TIF]

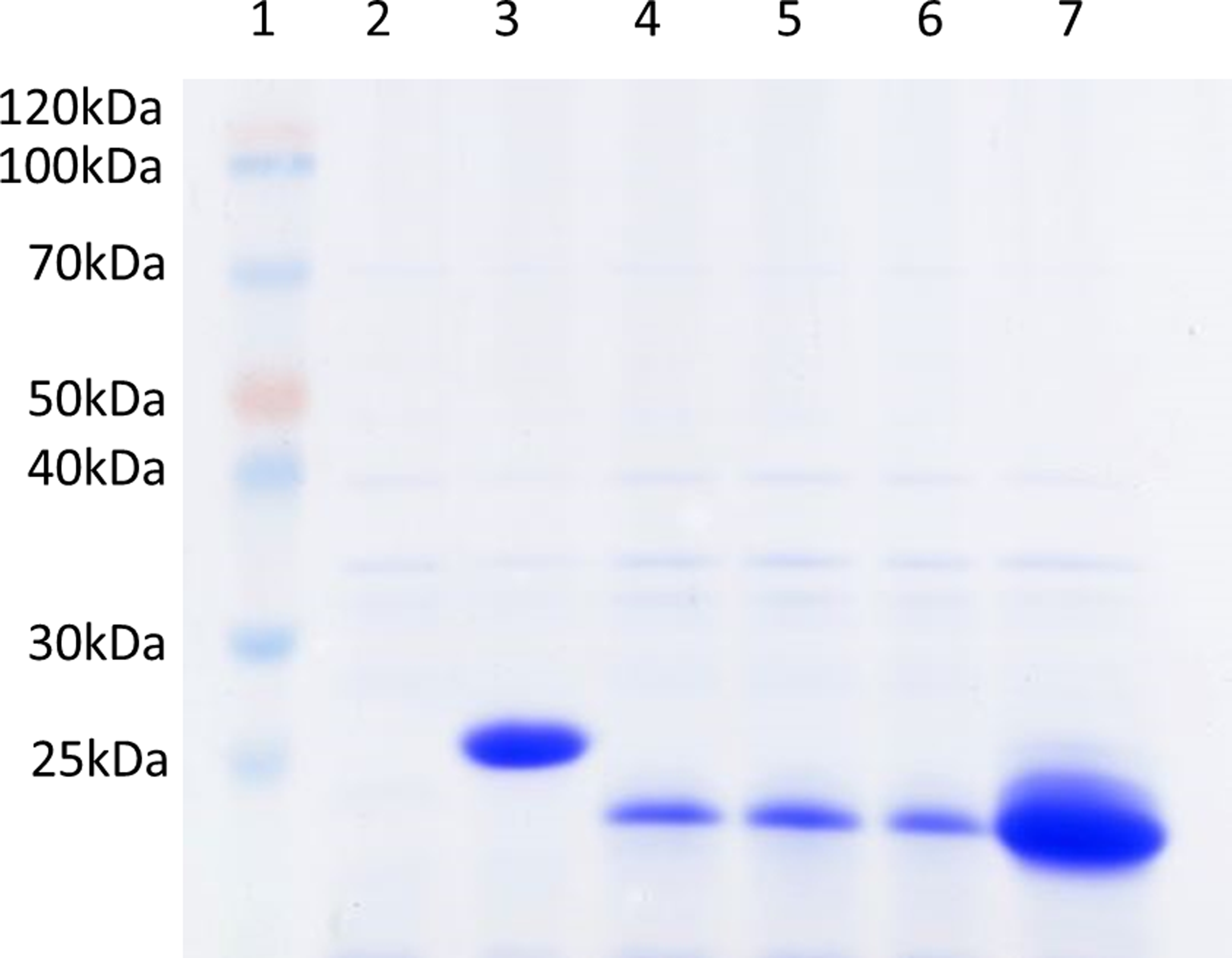

Supplement: Figure S3 — SDS-PAGE of purified WZY2 and its truncated derivatives expressed in BL21. Proteins were loaded in the order of marker (lane 1), HIS (control vector, lane 2), WZY2 (lane 3), ΔK1 (lane 4), ΔK2 (lane 5), ΔYS (lane 6), and ΔK1K2 (lane 7). Protein sizes in kDa are shown to the left of the panel. [file Image_3.TIF]

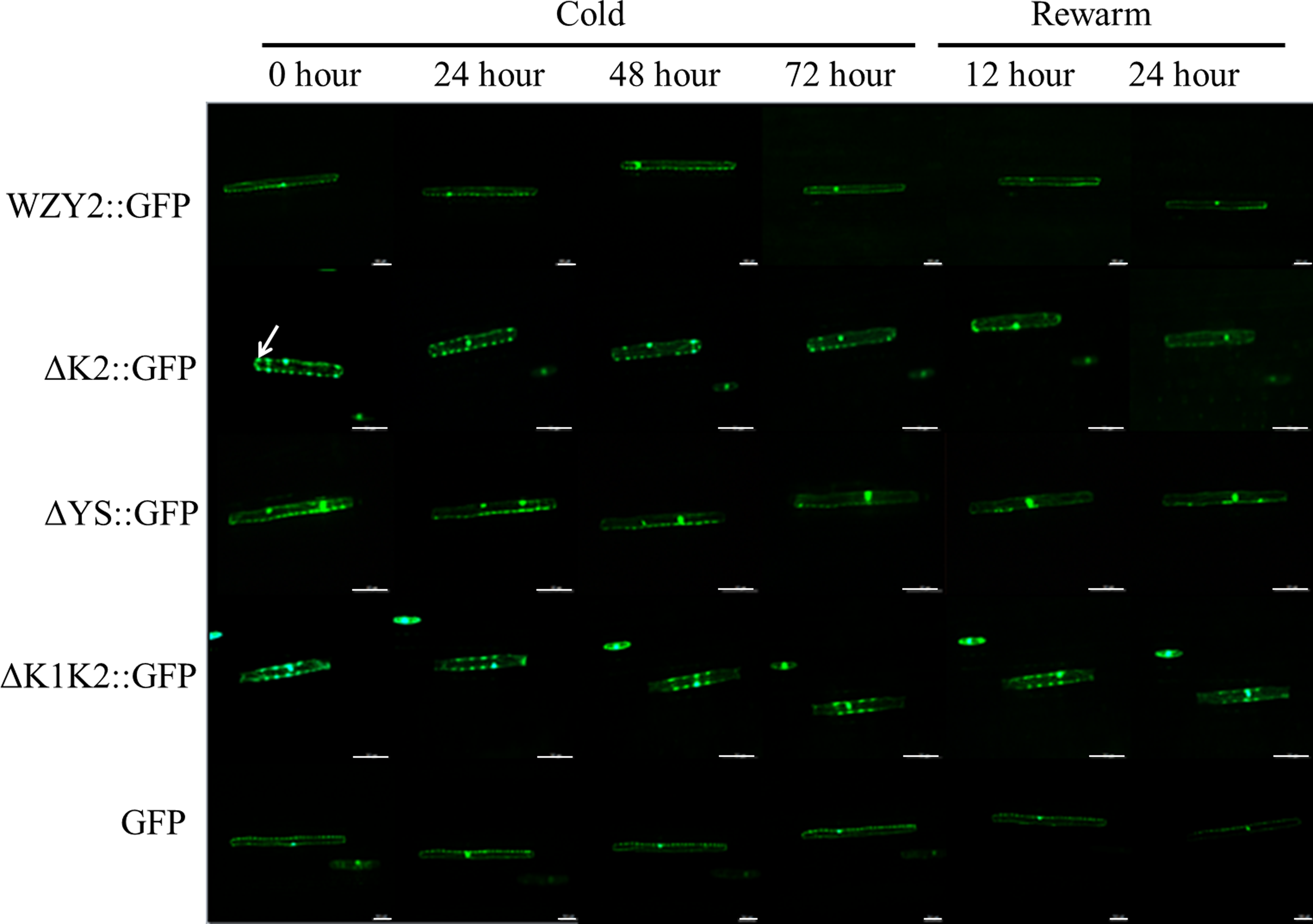

Supplement: Figure S4 — Subcellular localization of WZY2 (or ΔK2, ΔYS, ΔK1K2)::GFP fusion proteins. WZY2 (or ΔK2, ΔYS, ΔK1K2)::GFP fusion protein subcellular localization varied in wheat leaf blade epidermal cells when subjected for specified times to cold and rewarming treatments. The bar indicates 100 μm. Green fluorescence was dispersed in the nucleus, interspersed in the cytoplasm, and accumulated in spots (white arrow) near the plasma membrane. [file Image_4.TIF]
